# Correction: A model for regulation by SynGAP-α1 of binding of synaptic proteins to PDZ-domain 'Slots' in the postsynaptic density

**DOI:** 10.7554/eLife.22495

**Published:** 2016-10-19

**Authors:** Ward G Walkup, Tara L Mastro, Leslie T Schenker, Jost Vielmetter, Rebecca Hu, Ariella Iancu, Meera Reghunathan, Barry Dylan Bannon, Mary B Kennedy

Walkup IV WG, Mastro TL, Schenker LT, Vielmetter J, Hu R, Iancu A, Reghunathan M, Bannon BD, Kennedy MB. 2016. A model for regulation by SynGAP-α1 of binding of synaptic proteins to PDZ-domain 'Slots' in the postsynaptic density. *eLife* **5**:e16813. doi: 10.7554/eLife.16813.

Published 13, September 2016

The image in Figure 8A representing data for the assay in the absence of Ca^2+^ and calmodulin (-,-) was accidentally duplicated from the corresponding image in Figure 8B. The image in Figure 8A has now been corrected.

The data for these experiments were collected on a Typhoon Imager after exposure of gels to a phosphor screen that is then read by the Typhoon. The primary data was recorded digitally as numbers. We imported the numbers into Excel and then GraphPad Prism to make the graphs and do the statistical analyses shown in the figure. The images were generated separately from the graphs, and the accidental duplication in the originally published figure did not affect the numerical data or alter the conclusions of the study.

The corrected Figure 8 is shown here:

**Figure 1:**
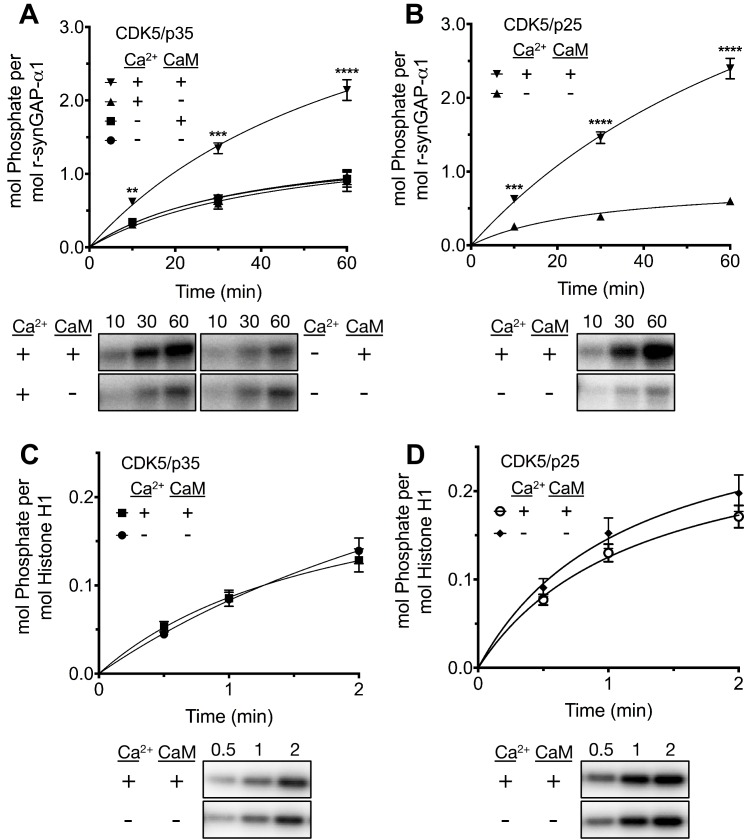

The originally published Figure 8 is also shown for reference:

**Figure 2:**
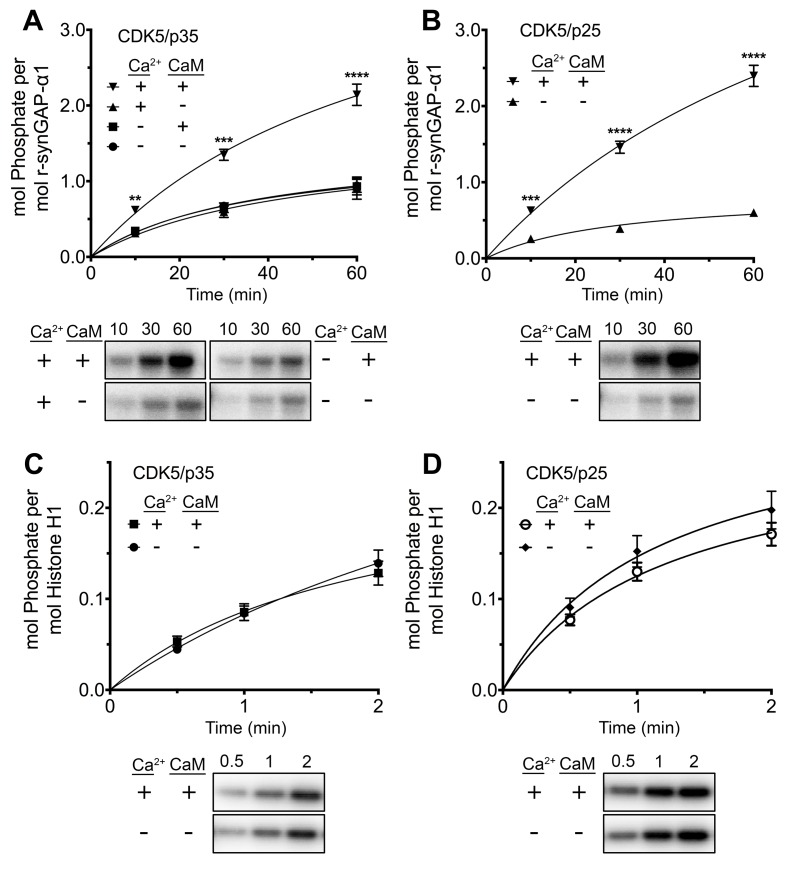

The article has been corrected accordingly.

